# Supplementary material for: Perinatal Whole Blood Zinc Status and Cytokines, Adipokines, and Other Immune Response Proteins
Source: Nutrients. 2019 Aug 22;11(9):1980. doi: 10.3390/nu11091980 (PMC6769600; doi:10.3390/nu11091980)
Supplement: Supplementary file 1 [file nutrients-11-01980-s001.pdf]

**Supplementary Table 1.** Relative change in mean neonatal levels of cytokines, adipokines and other proteins involved in the immune response with 95% confidence linked with every doubling in neonatal whole blood zinc content — results from models with correction for multiple testing.

| Outcome | Variable | Univariate Model | *p*-Value | Multivariate Model | *p*-Value |
|---|---|---|---|---|---|
| IL-1β | WB-Zinc content | 1.00 (0.85; 1.18) | 1.00 | 0.97 (0.82; 1.15) | 1.00 |
| IL-4 | WB-Zinc content | 0.99 (0.89; 1.09) | 1.00 | 0.97 (0.87; 1.08) | 1.00 |
| IL-6 | WB-Zinc content | 1.08 (0.94; 1.25) | 0.69 | 1.07 (0.92; 1.25) | 0.93 |
| IL-8 | WB-Zinc content | 0.98 (0.90; 1.07) | 1.00 | 1.02 (0.94; 1.10) | 1.00 |
| IL-10 | WB-Zinc content | 0.98 (0.76; 1.26) | 1.00 | 1.01 (0.79; 1.29) | 1.00 |
| IL-12 | WB-Zinc content | 1.08 (0.94; 1.25) | 0.76 | 1.03 (0.89; 1.17) | 1.00 |
| IFN-$\gamma$ | WB-Zinc content | 1.02 (0.89; 1.16) | 1.00 | 1.02 (0.88; 1.17) | 1.00 |
| TNF-$\alpha$ | WB-Zinc content | 1.07 (0.94; 1.21) | 0.86 | 1.01 (0.89; 1.14) | 1.00 |
| TGF-β | WB-Zinc content | 1.05 (0.94; 1.18) | 0.92 | 1.01 (0.90; 1.13) | 1.00 |
| Adiponectin | WB-Zinc content | 1.02 (0.93; 1.13) | 1.00 | 0.99 (0.91; 1.09) | 1.00 |
| Leptin | WB-Zinc content | 0.97 (0.87; 1.08) | 1.00 | 1.00 (0.90; 1.10) | 1.00 |
| CRP | WB-Zinc content | 0.91 (0.78; 1.06) | 0.62 | 0.97 (0.82; 1.14) | 1.00 |
| MBL | WB-Zinc content | 1.05 (0.89; 1.23) | 1.00 | 1.05 (0.88; 1.25) | 1.00 |
| sTREM-1 | WB-Zinc content | 0.99 (0.84; 1.17) | 1.00 | 0.95 (0.81; 1.11) | 0.99 |

Covariates included in the multivariate models are neonatal whole blood Zinc (WB-Zinc) content, case-status (cases are children that develops childhood type 1 diabetes (T1D), and controls do not develop T1D during childhood), birthweight and period. IL, interleukin; IFN-$\gamma$, interferon gamma; TNF-$\alpha$, tumor necrosis factor alpha; TGF-β, transforming growth factor beta; CRP, c-reactive protein; MBL, mannose-binding lectin; sTREM-1, soluble triggering receptor expressed on myeloid cells-1.
